# Influence of digital transformation on employee innovative behavior: roles of challenging appraisal, organizational culture support, and transformational leadership style

**DOI:** 10.3389/fpsyg.2025.1532977

**Published:** 2025-03-19

**Authors:** Jesna Lafortune Bindel Sibassaha, Jean Baptiste Bernard Pea-Assounga, Prince Dorian Rivel Bambi

**Affiliations:** ^1^School of Economics and Management, Jiangsu University of Science and Technology, Zhenjiang, China; ^2^School of Finance and Economics, Jiangsu University, Zhenjiang, China; ^3^Faculty of Economics, Marien Ngouabi University, Brazzaville, Republic of Congo

**Keywords:** challenging appraisal, digital transformation, employee innovative behavior, organizational culture support, structural equation modelling, transformational leadership style

## Abstract

**Introduction:**

This study investigates the relationships between digital transformation (DT), challenging appraisal (CA), organizational culture support (OCS), transformational leadership style (TLS), and employee innovative behavior (EINB) in Brazzaville’s banking sector. Grounded in Social Cognitive Theory and Self-Determination Theory, the research aims to provide insights into how these factors interact to influence innovation within organizations undergoing digital change.

**Methods:**

Data were collected from 280 employees working in the top five banks in Brazzaville. Structural Equation Modeling (SEM) was applied using SPSS 26 and SmartPLS to analyze the relationships among DT, CA, OCS, TLS, and EINB.

**Results:**

The findings reveal significant positive relationships between DT and CA, DT and EINB, and CA and EINB. Moreover, CA and OCS partially mediate the relationship between DT and EINB. While CA has a significant effect on EINB, TLS moderates the DT-EINB relationship by weakening its positive impact. Hypotheses regarding these relationships were largely confirmed, except for the interaction effect of DT and TLS on EINB.

**Discussion:**

These results highlight the crucial role of CA and OCS in facilitating the impact of digital transformation on employee innovation. Additionally, they suggest that higher levels of transformational leadership may not always strengthen innovation in digitally transforming organizations. The study provides valuable managerial implications for fostering an innovative workforce in the banking sector.

## 1 Introduction

In recent years, workplaces worldwide have experienced a profound evolution driven by the widespread adoption of digital technologies. This shift, known as ‘digital transformation,’ encompasses the integration of digital tools, processes, and strategies across organizational operations, with the aim of enhancing efficiency, competitiveness, and adaptability (Khin and Ho, 2019; Westerman et al., 2014). This phenomenon is particularly pertinent in Congo Brazzaville, where organizations are embracing digitalization to foster growth and development. Like many developing economies, Congo Brazzaville is undergoing a transformative phase in its technological journey (Pea-Assounga and Sibassaha, 2024), marked by initiatives to strengthen digital infrastructure, improve internet accessibility, and integrate digital solutions across sectors such as banking and government services (World Bank, 2021; Wu and Pea-Assounga, 2022). Despite challenges related to infrastructure and technology access disparities, the momentum toward digital transformation in Congo Brazzaville is palpable. As organizations in the country navigate this digital era, understanding the impact of technological advancements on employee behavior becomes essential. While prior research in Western contexts has hinted at a positive correlation between digital transformation initiatives and “employee innovative behavior” (Alieva and Powell, 2022; Bharadwaj et al., 2013), the specific dynamics of this relationship within the unique socio-cultural and organizational context of Congo Brazzaville remain largely unexplored.

Concurrently, the concept of ‘challenging appraisal’ has emerged prominently in organizational psychology literature as a mechanism for shaping employee innovative behavior. Challenging appraisal, defined as an evaluative process that not only assesses employee performance but also fosters a culture of continuous improvement by challenging individuals to exceed existing capabilities, warrants investigation within the realm of digital transformation (AlNuaimi et al., 2022; Hinings et al., 2018). Its potential mediating influence in the link between digital transformation and “employee innovative behavior” represents a critical area for exploration, particularly within the context of Congo Brazzaville. While existing studies have underscored the impact of digital transformation on organizational outcomes, such as increased efficiency, improved customer experiences, and enhanced competitive advantage (Bharadwaj et al., 2013; Westerman et al., 2014), the influence of digital transformation on employee behavior remains a critical area for exploration. For instance, research by Verhoef et al. (2021) demonstrated how digital transformation initiatives in the retail sector led to changes in employee roles and responsibilities, affecting job satisfaction and engagement. Similarly, a study by Selimović et al. (2021) highlighted how digital transformation in the financial industry influenced employee attitudes towards technology adoption and digital skill development, impacting overall organizational performance. This study aims to address this gap by investigating the influence of digital transformation initiatives on employee innovative behavior, while concurrently examining the mediating role of challenging appraisal practices and organizational culture support as well as the moderating effect of transformation leadership style within Congolese banks. The choice of Congolese banks as the focus of this study stems from several compelling reasons. Firstly, the banking sector in Congo Brazzaville represents a significant segment of the country’s economy, playing a crucial role in driving economic growth, facilitating financial transactions, and fostering investment and development (Opoko Apendi et al., 2017; Pea-Assounga et al., 2017). Therefore, understanding how digital transformation initiatives impact employee innovative behavior within Congolese banks holds strategic importance for both the banking sector and the broader economy. Secondly, the banking and financial sectors are undergoing fast digital transformation globally, and this trend is also evident in Congo Brazzaville. Banks in Congo Brazzaville are progressively integrating digital technologies to streamline their operations, improve customer experiences, and maintain competitiveness in the era of digitalization (Pea-Assounga et al., 2024). By focusing on Congolese banks, this study can provide insights into the unique challenges and opportunities faced by organizations in the banking sector in embracing digital transformation and fostering innovation among employees. Moreover, studying digital transformation and employee innovative behavior within Congolese banks offers a specific and tangible context for research, allowing for a deeper understanding of the complexities and nuances involved. By examining real-world practices and experiences within Congolese banks, this study can generate practical implications and recommendations that are directly relevant to the challenges and opportunities faced by organizations in the banking sector in Congo Brazzaville. By shedding light on these interconnected dynamics, this research seeks to contribute empirical insights that can inform organizational strategies aimed at leveraging digitalization to drive employee innovation in Congo Brazzaville. In addition to challenging appraisal, organizational culture support represents another vital determinant of employee innovative behavior in the context of digital transformation. Organizational culture, defined as the shared values, beliefs, and norms that shape behavior within an organization, can either facilitate or impede innovation. In digitally transformed organizations, fostering a culture that values experimentation, risk-taking, and creativity is essential for driving innovation (Guinan et al., 2019). Therefore, this study also aims to explore how organizational culture support acts as an intermediary factor in the connection between digital transformation and employees’ innovative behavior. Furthermore, the study explores the moderating effect of transformational leadership style on the relationship between digital transformation and employee innovative behavior. Transformational leaders are characterized by their ability to inspire and motivate employees, encourage innovation, and drive organizational change. In the context of digital transformation, transformational leadership may serve to amplify the effects of digital initiatives on employee behavior by providing vision, direction, and support (Tagscherer and Carbon, 2023). Therefore, this study seeks to examine how transformational leadership style influences the strength and direction of the relationship between digital transformation and employee innovative behavior.

Digital transformation in Congo Brazzaville is a multifaceted process involving the usage of digital technologies to drive societal, economic, and governmental advancements. Despite challenges in access and technology investment, significant progress has been made across various sectors. Telecommunications have witnessed substantial growth, facilitating communication, information access, and e-commerce (Pea-Assounga and Yao, 2021; World Bank, 2018). E-Government initiatives aim to enhance efficiency and transparency, while education increasingly integrates technology to improve learning outcomes (World Bank, 2018). The adoption of digital banking and fintech services is rising, promoting financial inclusion and facilitating transactions (Pea-Assounga and Wu, 2022; Wu and Pea-Assounga, 2022). Additionally, an emerging start-up ecosystem is fostering innovation and economic growth in sectors like fintech, healthcare, agriculture, and e-commerce (Pea-Assounga et al., 2024). Despite challenges such as limited internet connectivity and digital literacy, Congo Brazzaville is poised for further technological advancements with increasing investment and prioritization. The banking sector in Congo Brazzaville is experiencing a notable digital transformation, characterized by the introduction of online and mobile banking services, mobile money, and digital payment platforms (Pea-Assounga et al., 2024; World Bank, 2021). These initiatives aim to enhance accessibility, efficiency, and financial inclusion, albeit facing challenges like infrastructure limitations and cybersecurity concerns. Sustained investment, collaborations, and regulatory support are crucial for further catalyzing this transformation.

The main objective of this research is to evaluate the effect of digital transformation on employee innovative behavior and the mediating effect of challenging appraisal and organizational culture support as well as the moderating effect of Transformational Leadership Style. Specific objectives aim to: “Evaluate the effect of digital transformation on employee innovative behavior.” Examine the mediating effect of challenging appraisal and organizational culture support. Assess the moderating effect of transformational leadership style. Analyze the links between digital transformation, challenging appraisal, organizational culture support, and employee innovative behavior. The study addresses questions concerning the effect of digital transformation on employee innovative behavior (“*What is the effect of digital transformation on employee behavior?”*), the relationship between digital transformation and challenging appraisal (“*What is the relationship between digital transformation and challenging appraisal?”*), the influence of challenging appraisal on employee innovative behavior (*How does challenging appraisal influence employee behavior?*), and the potential mediation effects of challenging appraisal and organizational culture support (*Do challenging appraisal and organizational culture support mediate the relationship between digital transformation and employee innovative behavior?*).

Investigating the effect of digital transformation on employee innovative behavior, with a focus on the mediating role of challenging appraisal and organizational culture support, holds significant importance across several dimensions. Firstly, understanding how digital transformation influences employee behavior is crucial for organizational decision-making in a rapidly evolving digital landscape. Insights from this study can inform strategic decisions related to technology adoption and organizational culture, ultimately enhancing organizational effectiveness and competitiveness. Secondly, fostering a culture of innovation is vital for organizational growth, and this study offers practical implications for enhancing employee engagement and performance through effective challenging appraisal practices and Transformational Leadership Style. Thirdly, the study contributes to advancing theoretical frameworks in organizational behavior and innovation management, enriching existing perspectives and providing avenues for future research. This study innovatively bridges digital transformation and employee behavior domains by exploring how digital initiatives influence “employee innovative behavior.” By concentrating on the mediating effect of challenging appraisal, the study offers insights into the mechanisms shaping employee responses to digital transformation. Furthermore, the study provides methodological innovation by employing “Structural Equation Modeling (SEM)” to analyze complex models comprehensively, contributing to a more holistic understanding of the relationships among constructs.

This research is structured as follows: Section 1 presents the research topic and objectives, Section 2 reviews the theoretical foundations and relevant literature, Section 3 outlines the methodology, Section 4 presents the research findings, Section 5 discusses the results, and Section 6 addresses limitations and suggests directions for further research.

## 2 The study theoretical foundation

Social Cognitive Theory (SCT), proposed by Bandura (1991) offers insights into how digital transformation influences challenging appraisal among employees. This theory underscores the significance of observational learning, self-efficacy, and motivation in shaping individual behavior. Observational learning suggests that employees observe and learn from others’ innovative behaviors, influencing their own. Additionally, the challenging appraisal can enhance employees’ self-efficacy beliefs, leading to greater motivation for innovative activities. Studies by Han and Xia (2020) and Trenerry et al. Yang et al. (2021) support these assertions, showing that observing innovative behaviors and having digital self-efficacy positively impact employees’ engagement in innovation initiatives. Furthermore, SCT highlights the reciprocal interaction between individuals and their environment, indicating that organizational climate and leadership practices, such as transformational leadership, significantly influence employees’ innovative behaviors. Research by Hoang et al. (2022) corroborates this, highlighting the role of transformational leadership in shaping employees’ perceptions of digital transformation efforts, thereby influencing their innovative behaviors. On the other hand, the “Self-Determination Theory (SDT)” proposed by Deci and Ryan (1985), offers insights into the link between challenging appraisal and employee innovative behavior. SDT posits that individuals are motivated by psychological needs for autonomy, competence, and relatedness. In this regard, challenging appraisal aligns with these needs by providing autonomy in problem-solving, fostering competence through overcoming challenges and promoting relatedness by encouraging collaboration. Empirical support from research by Van Den Broeck et al. (2016) and Deci et al. (2017) strengthens these claims, demonstrating that autonomy support and challenging tasks positively influence employees’ intrinsic motivation and innovative behavior. Moreover, SDT emphasizes the role of leaders and organizational practices in creating an environment that supports autonomy, competence, and relatedness, highlighting the importance of transformational leadership in fostering innovative behavior in digitally transformed workplaces. Additionally, a study by Nazir et al. (2019) found a significant positive relationship between employees’ perceived organizational culture support for innovation and their innovative behavior, suggesting that a supportive organizational culture fosters innovation among employees in digitally transformed environments. These findings further underscore the importance of organizational context in shaping employee behavior in the digital era.

### 2.1 Empirical literature review

In this section, the scholar compares theoretical reviews of literature with the results of other research works by analyzing the notions of other scholars in some manner in reference to the subject at hand.

Numerous investigations have delved into the correlation between digital transformation and employee innovation. For instance, a study conducted by Westerman et al. (2014) found that organizations undergoing digital transformation experienced higher levels of innovation, as employees were empowered to leverage digital tools and technologies to generate new ideas and solutions. Similarly, a study by Hanelt et al. (2021) demonstrated a positive linkage between digital capabilities and innovation outcomes in organizations. These findings suggest that digital transformation initiatives create an environment conducive to fostering employee innovation. Research by Tarafdar and Tanriverdi (2018) investigated the influence of “digital transformation” on employee creativity and innovation. The study found that organizations with advanced digital capabilities exhibited higher levels of employee creativity and innovation. Specifically, digital technologies provide employees with access to diverse information, collaboration tools, and creative platforms, enabling them to generate and implement innovative ideas more effectively. Moreover, research by AlDhaheri et al. (2023) examined the “factors influencing employee innovation behavior in the context of digital transformation.” The findings revealed that organizational support, leadership encouragement, and access to digital resources were significant predictors of employee innovation. This suggests that organizational factors, including appraisal practices, play a crucial role in fostering employee innovation within digitally transformed environments.

The role of challenging appraisal in shaping employee behavior has also been investigated in empirical studies. For instance, Harari et al. (2018) conducted a meta-analysis of research on performance appraisal and employee creativity. The results revealed a positive link between challenging appraisal practices (e.g., setting ambitious goals, and providing constructive feedback) and employee creative performance. Employees who received challenging appraisals were more likely to engage in creative problem-solving and generate innovative solutions. Research by Speer et al. (2020) found that employees who received challenging performance appraisals were more likely to demonstrate higher degrees of engagement and motivation. Similarly, a study by Zia et al. (2022) showed that challenging feedback from supervisors positively influenced employee learning and development. These findings suggest that challenging appraisal practices can impact employee behavior and performance outcomes.

While empirical research specifically examining the mediating effect of challenging appraisal in the linkage between digital transformation and employee innovative behavior is limited, studies have explored related constructs. For example, a study by Zhang X. et al. (2023) investigated “the mediating effect of performance feedback on the relationship between digital transformation and employee performance.” The results indicated that performance feedback mediated the relationship, suggesting that appraisal processes may play a significant role in translating digital transformation efforts into employee outcomes. Similarly, research by Liu and Su (2022) found that organizational support mediated the link between digital capabilities and employee innovative behavior. These findings underscore the importance of considering appraisal processes as potential mediators in the context of digital transformation and employee behavior. While not directly focused on digital transformation, research by Yan et al. (2022) explored the mediating role of challenging appraisal in the context of organizational change. The study found that challenging performance feedback mediated the linkage between change-related communication and employee attitudes toward change. Employees who received challenging feedback perceived organizational change initiatives more positively and were more likely to actively participate in the change process, suggesting that challenging appraisal practices can facilitate organizational change efforts.

Recent studies have highlighted the importance of integrating digital transformation initiatives with appraisal practices to enhance employee outcomes. For example, research by Yang et al. (2021) examined the effects of digital performance appraisal systems on employee motivation and job satisfaction. The findings indicated that digital appraisal systems positively influenced employee perceptions of fairness and transparency, leading to higher degrees of motivation and job satisfaction. This suggests that digital transformation initiatives in the realm of performance appraisal can contribute to fostering employee engagement and innovative behavior. In addition, research by Weber et al. (2022) investigated how digital transformation initiatives interact with challenging performance appraisal to influence employee job satisfaction and performance. The results indicated that the combination of digital capabilities and challenging appraisal practices had a synergistic effect on employee outcomes, leading to higher levels of job satisfaction and performance.

### 2.2 Conceptual framework and hypothesis development

#### 2.2.1 Theoretical model

**Figure 1:**
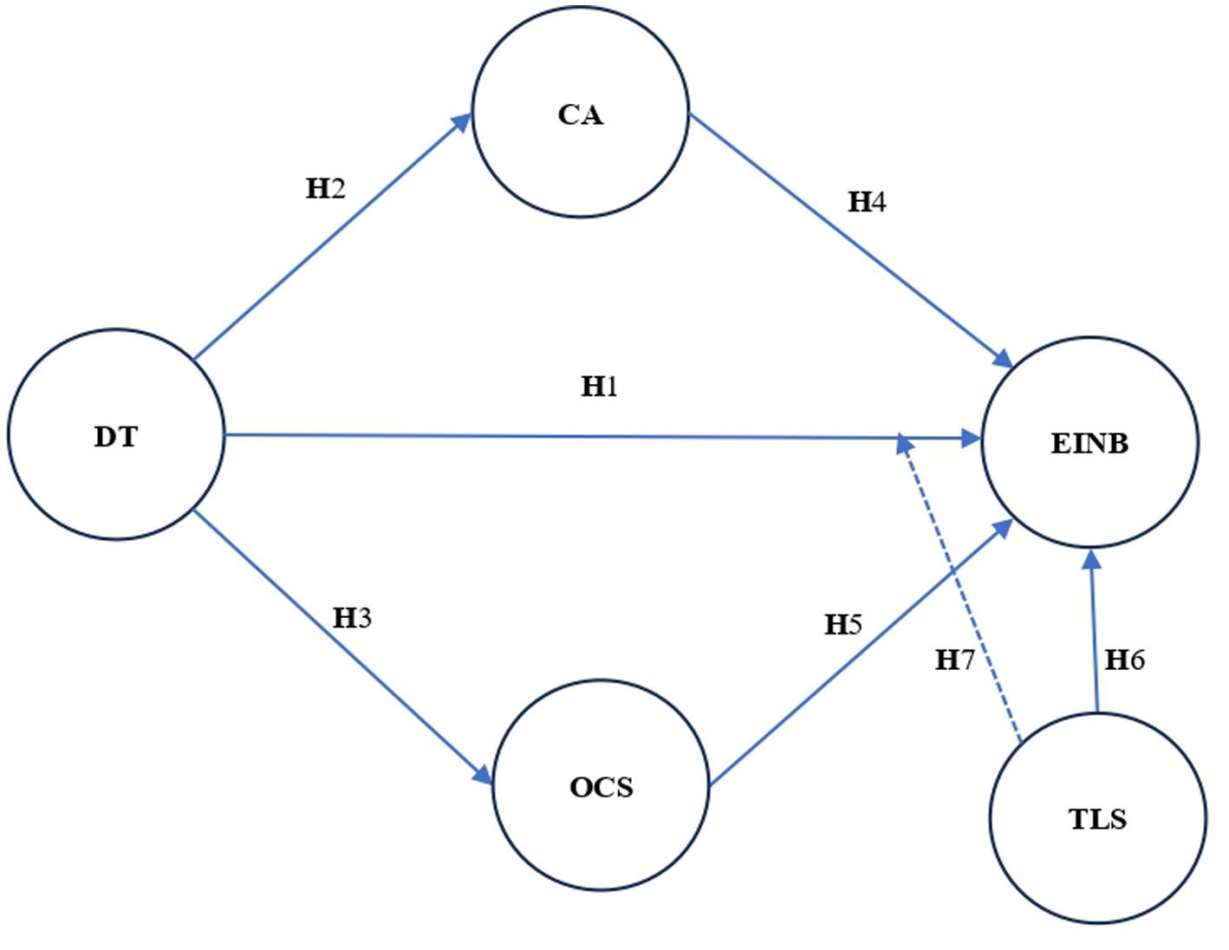
Theoretical framework.

The theoretical model outlined by Shields and Rangarajan (2013), visually illustrates the connection between dependent, mediating, and independent variables. In this research, the dependent construct is employee innovative behavior (EINB), while the mediators are challenging appraisal (CA), and organizational culture support (OCS). Finally, the external constructs are digital transformation (DT) and transformation leadership style (TLS). This work has utilized a theoretical framework, depicted in Figure 1 to elucidate employee innovative behavior.

#### 2.2.2 Hypothesis development

##### 2.2.2.1 Digital transformation and employee innovative behavior

From the literature review, empirical studies have shown that digital transformation, by providing employees with improved access to data and information through advanced technological tools, positively correlates with increased innovative behavior (Bharadwaj et al., 2013). This increased access facilitates idea generation, problem-solving, and knowledge sharing among employees, fostering a culture of innovation within the organization. An investigation by Iivari et al. (2020) found a strong relationship between digital collaboration platforms and employee innovative behavior. These platforms, such as online collaboration tools and project management software, enable real-time communication and idea sharing among teams, leading to enhanced innovation by leveraging collective intelligence. Other studies suggest that employees’ adaptability to technological changes induced by digital transformation positively influences their innovative behavior (Galliers and Leidner, 2014). Employees who embrace and effectively utilize new digital tools and technologies are more likely to exhibit innovative behaviors in problem-solving and creative ideation. Research by Scuotto et al. (2023) emphasizes the importance of digital skill development programs in fostering employee innovation. Digital transformation initiatives that incorporate training and upskilling opportunities for employees to leverage technological tools tend to result in higher levels of innovative behavior. Organizational support for digital transformation initiatives significantly impacts employee innovative behavior. A study by Westerman et al. (2014) highlighted that organizational cultures that actively encourage and reward digital experimentation and innovation tend to see higher levels of innovative behavior among employees involved in such initiatives. Another study by Shin et al. (2023) found a positive link between employees’ engagement in digital transformation projects and their innovative behavior. Actively involving employees in digital initiatives and decision-making processes fosters a sense of ownership and empowerment, leading to increased innovative contributions. Research conducted by Huu (2023) demonstrated a positive association between the use of digital technologies for problem-solving purposes and employee innovative behavior. Digital tools that aid in identifying, analyzing, and solving complex problems tend to stimulate creative thinking and innovation among employees. These empirical findings collectively support the notion that digital transformation initiatives within organizations significantly influence and contribute to the innovative behavior exhibited by employees across various domains, emphasizing the need to further explore this relationship in different contexts and industries.

*H1. Digital Transformation has a positive effect on Employee Innovative Behavior.*

##### 2.2.2.2 Digital transformation and challenging appraisal

There is scarce empirical evidence elucidating the correlation between digital transformation and challenging appraisal practices. Research by Saeed et al. (2023) indicates that digital transformation initiatives significantly impact organizational culture. Organizations undergoing digital transformation tend to exhibit a more open, flexible, and growth-oriented culture. This cultural shift often aligns with challenging appraisal practices, where performance evaluations focus on continuous improvement and development rather than just assessment. Studies by Mihai et al. (2022) highlight how digital transformation enables the implementation of real-time performance metrics and feedback mechanisms. These tools facilitate more dynamic and data-driven appraisal systems, allowing for more personalized and challenging feedback tailored to individual employee performance and growth areas. The investigation by Trenerry et al. (2021) explores the link between digital transformation and agile work methodologies. Agile frameworks often incorporate challenging appraisal as a core element, fostering an environment where frequent feedback, experimentation, and learning are valued. Digital transformation initiatives often align with these agile practices, reinforcing challenging appraisal processes. Studies suggest that digital tools, integrated as part of the digital transformation process, provide avenues for continuous learning and development (Braojos et al., 2024). These tools enable ongoing skill enhancement, performance tracking, and personalized development plans, contributing to a culture of challenging appraisal focused on growth and skill advancement. Research by Ye et al. (2024) demonstrates a positive linkage between employee engagement in digital transformation efforts and the effectiveness of challenging appraisal systems. Actively involving employees in the digitalization process fosters a culture of collaboration, learning, and receptiveness to challenging feedback. The usage of digital platforms for performance evaluation and feedback delivery has been shown to enhance challenging appraisal practices (Nudurupati et al., 2021). These platforms facilitate transparent, timely, and constructive feedback loops, crucial for fostering a culture of continuous improvement.

These empirical findings collectively suggest a strong relationship between digital transformation initiatives and the adoption or reinforcement of challenging appraisal practices within organizations. Digital tools and cultural shifts associated with digital transformation often facilitate or complement challenging appraisal processes, emphasizing continuous improvement, skill development, and growth-oriented performance evaluation.

*H2. Digital Transformation may influence positively Challenging Appraisal.*

##### 2.2.2.3 Digital transformation and organizational culture support

Empirical research suggests a strong linkage between digital transformation (DT) and organizational culture support. The work by Kim et al. (2022) investigated the “impact of digital transformation on organizational culture in the context of South Korean companies.” Their findings revealed that successful digital transformation initiatives were associated with a cultural shift towards agility, innovation, and customer-centricity. Organizations that effectively leveraged digital technologies to streamline processes and enhance customer experiences tended to develop a culture that embraced change and experimentation.

Similarly, research by Singh et al. (2021) explored “the relationship between digital transformation and organizational culture support in Indian firms.” They found that organizations that prioritized digitalization efforts experienced significant improvements in their culture, characterized by enhanced communication, collaboration, and adaptability. Digital technologies played a pivotal role in facilitating this cultural transformation by empowering employees to work more efficiently and creatively. Additionally, Ghafoori et al. (2024) discuss the importance of organizational culture in supporting digital transformation, suggesting that a culture of agility, experimentation, and learning, fostered by challenging appraisal practices, can mediate the link between digital transformation and employee behaviors.

Furthermore, a study by Zhang G. et al. (2023) examined the influence of organizational culture on the success of digital transformation projects in Chinese companies. Their research revealed that organizations with a culture that values innovation, learning, and risk-taking were more likely to achieve positive outcomes from their digital initiatives. Cultivating a culture of openness and experimentation was identified as a critical factor in overcoming resistance to change and driving successful digital transformation. Moreover, research by Zoppelletto et al. (2023) investigated the relationship between digital transformation and organizational culture support in the context of “small and medium-sized enterprises (SMEs).” They found that SMEs that embraced digital technologies tended to develop a culture that prioritized collaboration, transparency, and employee empowerment. These cultural attributes were instrumental in fostering innovation and agility, enabling SMEs to adapt to rapidly changing market conditions.

Overall, recent empirical evidence reaffirms the significant link between digital transformation and organizational culture support. Organizations that effectively integrate digital technologies into their operations are more likely to cultivate a culture that promotes innovation, collaboration, and adaptability, ultimately driving success in today’s dynamic business environment.

*H3. Digital Transformation may influence positively Organizational Culture Support.*

##### 2.2.2.4 Challenging appraisal and employee innovative behavior

Research by Ashford et al. (2016) suggests that high-quality feedback, which is a core component of challenging appraisal, positively influences employee innovative behavior. Constructive and specific feedback provided during challenging appraisal processes encourages employees to explore new ideas and take innovative initiatives. Studies by Miao et al. (2023) emphasize the link between a growth mindset encouraged by challenging appraisal and employee innovation. When employees perceive performance evaluations as opportunities for growth rather than mere assessments, they are more inclined to exhibit innovative behaviors and seek out new solutions. Research by Liu et al. (2022) found that setting challenging yet attainable goals during performance appraisals stimulates employee creativity. Challenging appraisal practices that involve setting ambitious but achievable objectives can motivate employees to think innovatively while striving to meet these goals. Studies by Van Dyck et al. (2005) suggest that an organizational culture emphasizing frequent and challenging feedback fosters a conducive environment for idea generation. Employees who receive consistent and challenging feedback are more likely to engage in creative problem-solving and innovative thinking. Research by Zhou et al. (2014) highlights that challenging appraisal processes that recognize and reward calculated risk-taking behavior tend to stimulate employee innovation. Employees are more willing to take risks and experiment with novel ideas when such behavior is acknowledged and valued during performance evaluations. Studies by Miron-Spektor et al. (2011) suggest a positive relationship between employee engagement in challenging appraisal practices and their inclination toward innovative behaviors. Actively involving employees in challenging performance appraisal discussions and goal-setting processes fosters a sense of ownership and motivation to innovate. These empirical findings collectively indicate a robust relationship between challenging appraisal practices and employee innovative behavior. Challenging appraisal processes that emphasize high-quality feedback, growth-oriented mindsets, challenging goals, recognition of risk-taking, and employee engagement tend to stimulate and nurture a culture of innovation within organizations.

*H4. Challenging Appraisal can have a positive effect on Employee Innovative Behavior.*

##### 2.2.2.5 Organizational culture support and employee innovative behavior

Recent empirical evidence continues to support the notion of a strong relationship between organizational culture support and employee innovative behavior. Research by Nguyen et al. (2019) conducted in Vietnam’s IT firms found that a supportive organizational culture characterized by openness, collaboration, and risk-taking positively influenced employees’ willingness to innovate. Similarly, a study by Liu et al. (2019) in the technology sector revealed that organizations with a culture emphasizing experimentation and learning experienced higher levels of employee creativity and innovation.

Furthermore, a cross-national study by Lee (2020) examined the impact of organizational culture on innovative behavior in South Korean and U.S. firms. They found that a supportive culture that values creativity and provides resources for innovation significantly predicted employees’ innovative behaviors in both contexts.

In another study, Khan et al. (2020) explored the role of leadership in shaping organizational culture and its impact on innovation. Their findings indicated that “transformational leadership behaviors,” such as inspirational motivation and intellectual stimulation, are positively associated with a culture of innovation, which, in turn, fostered employee innovative behavior. Moreover, research by Tsui et al. (2006) investigated the “influence of organizational culture dimensions, including adaptability and involvement, on employee innovation in Chinese manufacturing firms.” The results revealed a positive and significant relationship between these cultural dimensions and employee innovative behavior, highlighting the importance of fostering a culture that encourages experimentation and employee involvement in decision-making processes.

Overall, recent empirical studies provide robust evidence supporting the idea that organizational culture support has a significant role in stimulating and nurturing employee innovative behavior across various industries and cultural contexts.

*H5. Organizational Culture Support influences positively Employee Innovative Behavior.*

##### 2.2.2.6 The direct and moderating effects of transformation leadership style on employee innovative behavior and the link between DT and EINB

Recent empirical research has shed light on both the direct and moderating impacts of transformational leadership style on employee innovative behavior, along with its function in the correlation between digital transformation and “employee innovative behavior.”

In a study by Zhu et al. (2022), conducted in a Chinese manufacturing context, transformational leadership is found to directly influence employee innovative behavior positively. The research highlighted that leaders who exhibit transformational qualities, such as inspiring vision, individualized consideration, intellectual stimulation, and supportive behavior, tend to foster a conducive environment for innovation among employees. This direct relationship underscores the importance of leadership in driving employee creativity and innovation within organizations. Furthermore, this study also explored the moderating effect of transformational leadership in the relationship between digital transformation and employee innovative behavior. Results indicated that transformational leadership strengthens the positive influence of digital transformation on “employee innovative behavior.” Leaders who effectively communicate the vision of digital transformation, provide support, and empower employees to embrace technological changes can enhance the innovation potential of their workforce (Ertiö et al., 2024). This moderating effect suggests that transformational leadership amplifies the benefits of digital transformation initiatives on employee innovation outcomes. Similarly, research by Odugbesan et al. (2023) investigated the linkage between transformational leadership, digital transformation, and employee innovative behavior in the context of information technology (IT) firms. The study revealed that transformational leadership influences positively both digital transformation efforts and employee innovative behavior. Moreover, “transformational leadership” significantly moderated the link between digital transformation and employee innovative behavior, indicating that strong leadership enhances the effectiveness of digital initiatives in driving innovation within IT firms.

These empirical findings underscore the critical role of transformational leadership in fostering employee innovative behavior and facilitating the success of digital transformation initiatives. Leaders who exhibit transformational qualities can motivate and inspire employees to embrace change, adapt to new technologies, and contribute creatively to organizational goals. Moreover, transformational leadership serves as a key driver in maximizing the influence of digital transformation on employee innovation, highlighting its significance in navigating organizational change and fostering a culture of continuous improvement and innovation.

*H6. Transformational leadership Style positively influences Employee Innovative Behavior.*

*H7. Transformation Leadership Style moderates the link between Digital Transformation and Employee Innovative Behavior.*

##### 2.2.2.7 The “mediating effect” of CA on the linkage between DT and EINB

The investigations specifically concentrating on the mediating effect of challenging appraisal on the link between digital transformation and employee innovative behavior are somewhat limited but emerging. While direct studies on the mediating role are limited, an investigation by Westerman et al. (2014) highlights that challenging appraisal practices play a crucial role in enabling employees to effectively leverage digital transformation initiatives. The study indicates that an organizational culture emphasizing continuous improvement and challenging appraisal aligns well with the requirements for successful digital transformation efforts. This alignment hints at the potential mediating role of challenging appraisal in facilitating the link between digital transformation and employee behavior. Studies by Scuotto et al. (2023) highlight that employee-centric feedback systems integrated into digital transformation initiatives are essential for fostering a culture of innovation. These feedback mechanisms, part of the challenging appraisal, allow for personalized feedback and developmental guidance, thereby influencing employee behaviors and potentially mediating the influence of digital transformation on innovation. Ghafoori et al. (2024) discuss the importance of organizational culture in supporting digital transformation. They suggest that a culture of agility, experimentation, and learning, fostered by challenging appraisal practices, can mediate the relationship between digital transformation and employee behaviors. Such a culture promotes the acceptance and the usage of “digital tools,” enhancing the likelihood of innovation among employees. A study by Saeed et al. (2023) expressed that digital transformation initiatives often coincide with skill development programs. Challenging appraisal practices aligned with digital skill enhancement may act as a mediator by fostering an environment where employees are motivated to apply newly acquired digital skills toward innovative activities. Another study by AlNuaimi et al. (2022) found that employee engagement in digital transformation initiatives positively influences the effectiveness of performance appraisal systems. Actively involving employees in digital initiatives and aligning challenging appraisal with these efforts might create a conducive environment for fostering innovative behaviors. While direct empirical evidence explicitly establishing challenging appraisal as a mediator between digital transformation and employee innovative behavior might be limited, these studies collectively hint at the potential mediating role of challenging appraisal practices in influencing employee behavior in the context of digital transformation initiatives. Further empirical research explicitly exploring this mediating relationship within the framework of digital transformation in various organizational contexts is warranted to solidify these associations.

*H8. Challenging Appraisal may mediate the link between DT and EINB.*

##### 2.2.2.8 The mediating role of OCS on the link between DT and EINB

Recent empirical studies have shed light on the mediating role of organizational culture support in the relationship between digital transformation and employee innovative behavior. A study by Kim et al. (2022) assessed the “mediating effect of organizational culture support on the link between digital transformation initiatives and employee innovative behavior in South Korean firms.” Their findings revealed that a supportive organizational culture, characterized by openness to change, collaboration, and risk-taking, significantly mediated the relationship between digital transformation efforts and employee innovation. Organizations that fostered a culture of experimentation, learning, and knowledge sharing were better equipped to harness the potential of digital technologies to drive innovation among their workforce. Moreover, research by Liang and Li (2023) investigated the mediating role of organizational culture support in the context of digital transformation in Chinese technology companies. They found that a culture that values agility, adaptability, and continuous improvement acted as a significant mediator in the link between digital transformation initiatives and employee innovative behavior. Organizations that prioritized cultural elements such as trust, autonomy, and recognition of employee contributions were able to create an environment conducive to innovation, enabling employees to embrace digital changes and leverage technology for creative problem-solving and idea generation. Furthermore, an investigation by Bagga et al. (2023) explored “the mediating role of organizational culture support on the relationship between digital transformation and employee innovative behavior in Indian multinational corporations (MNCs).” Their research highlighted the crucial role of a supportive organizational culture in facilitating the integration of digital innovation and technologies into everyday work practices and stimulating employee creativity and innovation. Organizations that cultivated a culture of experimentation, collaboration, and empowerment were more successful in driving innovative behaviors among their employees in the setting of digital transformation. Additionally, research by Chen et al. (2024). investigated the mediating role of organizational culture support in the Chinese manufacturing sector, specifically focusing on the influence of digital transformation on employee innovative behavior in Chinese firms. Their findings demonstrated that organizational culture support, characterized by a commitment to customer-centricity, agility, and continuous learning, mediated the link between digital transformation initiatives and employee innovation. Firms that fostered a culture of innovation and digital readiness were better able to capitalize on technological advancements to drive business innovation and enhance customer experiences (Sun et al., 2024).

*H9. Organizational Culture Support may mediate the link between DT and EINB.*

## 3 Methodology

### 3.1 Research design and context

This research uses a “quantitative research design” to empirically evaluate the relationships between digital transformation (DT), challenging appraisal (CA), organizational culture support (OCS), transformational leadership style (TLS), and employee innovative behavior (EINB). A cross-sectional survey was conducted to collect data from employees working in the top five (5) banks in Congo Brazzaville. These banks were strategically selected based on their market share dominance and their advanced implementation of digital transformation initiatives. The research setting is characterized by an evolving financial sector that has increasingly adopted digital technologies to enhance efficiency, customer experience, and competitive advantage. Employees from various functional departments (e.g., IT, customer service, operations, and management) were included to ensure a diverse and representative sample of perspectives on digital transformation and its effects on employee innovative behavior.

### 3.2 Sampling strategy and data collection

A random sampling technique was used to ensure fairness in participant selection, minimizing biases and enhancing the generalizability of the findings. A total of 330 structured surveys were distributed to employees across different hierarchical levels within the five banks. Prior to distribution, ethical approval and support from the organization’s management and HR departments were obtained to facilitate participant engagement and ensure compliance with ethical research standards. To enhance the validity and reliability of the instrument, a pilot study involving 30 respondents was conducted. Response from the pilot phase informed adjustments to improve clarity, wording, and relevance of certain items. The final survey instrument was then administered, and responses were collected over a four-week period. Out of the 330 distributed surveys, all were returned, yielding a 100% initial response rate. However, following a rigorous data cleaning process, 50 incomplete surveys were excluded, resulting in 280 complete and valid responses for analysis, representing an effective response rate of 85%.

### 3.3 Constructs measurements and source

The measurements utilized in this work were derived from previous research and modified to suit the current investigation. The measurements of digital transformation were adopted from Khin and Ho (2019), while the items for challenging appraisal were derived from Cavanaugh et al. (2000). The indicators for organizational culture support and transformational leadership style are obtained from Upadhyay and Kumar (2020) and Afsar and Umrani (2019), respectively. Finally, the items of employee innovative behavior were taken from (Scott and Bruce, 1994). In this research, a “Likert scale” ranging from one (1) denoting “strongly disagree” to five (5) indicating “strongly agree” was utilized.

### 3.4 Data analysis and statistical techniques

Data analysis was conducted using SmartPLS 3 and SPSS 26, following a structured multi-step approach. Descriptive statistics were used to analyze the sample characteristics, including means, standard deviations, skewness, and kurtosis, ensuring data normality. Reliability and validity testing included content validity, ensured through expert reviews and pilot testing; construct reliability, evaluated using “Composite Reliability (CR)” and “Cronbach’s Alpha (α)” to ensure internal consistency; convergent validity, assessed using “Average Variance Extracted (AVE)” to confirm that the items measure their respective constructs; and discriminant validity, examined using the Fornell-Larcker criterion to confirm that constructs are conceptually distinct. “Exploratory Factor Analysis (EFA)” and “Confirmatory Factor Analysis (CFA)” were conducted to validate the measurement model and ensure construct validity. Multicollinearity assessment was performed by calculating the “Variance Inflation Factor (VIF)” to detect potential collinearity issues among predictors. “Structural Equation Modeling (SEM)” was implemented in SmartPLS 3 to test the proposed hypotheses and assess direct, indirect, and moderating effects. Lastly, bootstrapping analysis was used to verify the significance of mediation and moderation effects with a 5,000 resampling approach.

The study adhered to “ethical research principles, including informed consent, confidentiality, and voluntary participation.” Participants were assured that their responses would remain anonymous and used solely for academic purposes. Furthermore, ethical approval was sought from relevant institutional review boards before data collection commenced. This methodological framework ensures the credibility, reliability, and validity of the study’s findings while providing a nuanced understanding of how digital transformation interacts with leadership, organizational support, and employee innovation in a rapidly evolving financial sector.

## 4 Findings

### 4.1 Demographic information

Table 1 presents the demographic information of the participants involved in the study. The table provides frequencies and percentages for various demographic variables. Regarding age distribution, the majority of participants fall into the 30–35 age group (29.3%), followed by the 36–40 age group (26.4%). A considerable portion of participants are also in the 24–29 age group (23.2%). The distribution of participants across gender shows a slight majority of males (54.3%) compared to females (45.7%). Regarding “education level,” undergraduates or bachelor’s degree holders make up the largest proportion (45.7%), followed by graduates with a master’s or Ph.D. degree (30.7%). Other education levels represent 23.6% of participants. Regarding departmental affiliation, the majority of participants are from customer service (36.1%), followed by marketing (27.1%). Finance and human resources departments constitute smaller proportions at 21.1 and 15.7%, respectively. Regarding work experience, participants with 4–6 years of experience represent the largest group (40.4%), followed by those with 7–9 years of experience (30.0%). Participants with less than or equal to 3 years of experience account for 16.0%, while those with 10 years or more of experience constitute 13.6% of the sample. Overall, the table provides a comprehensive overview of the demographic characteristics of the study participants, facilitating insights into the composition of the sample.

**Table 1:** Demographic profile of participants.

	Frequency	%
Age (in years)		
24–29	65	23.2
30–35	82	29.3
36–40	74	26.4
41 & above	59	21.1
Gender		
Male	152	54.3
Female	128	45.7
Education level		
Undergraduates/Bachelors	128	45.7
Graduates (Master or Ph.D.)	86	30.7
Others	66	23.6
Department		
Finance	59	21.1
Marketing	76	27.1
Customer Service	101	36.1
Human Resource	44	15.7
Work experience (in years)		
≤3	45	16.0
4–6	113	40.4
7–9	84	30.0
10 & above	38	13.6

Number of Participants = 280.

### 4.2 Data analysis

#### 4.2.1 Reliability and validity

**Figure 2:**
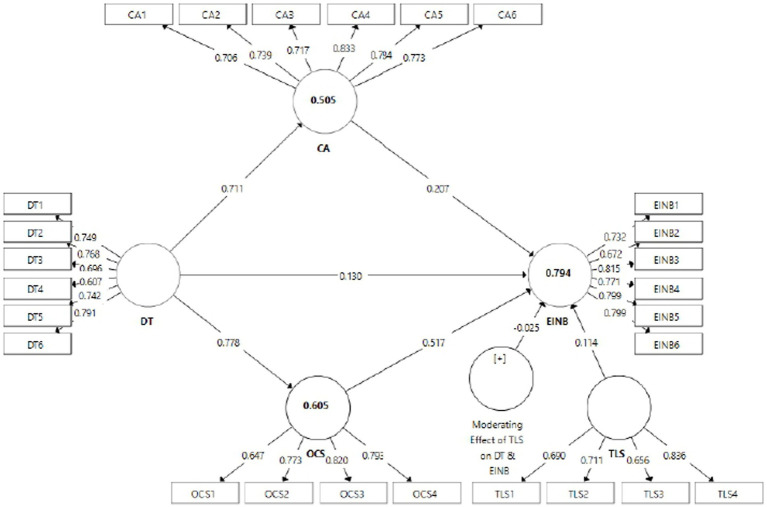
Structural equation modeling output from SmartPLS.

Hair Jr et al. (2023) outlined the primary criteria for evaluating the estimation model, commonly recognized as an external model, to ensure the validity and reliability of the employed constructs. To assess the constructs’ reliability, both Cronbach’s Alpha and composite reliability values have been examined. The analysis indicated that “Cronbach’s Alpha” spanned from 0.709 to 0.859, exceeding the threshold standard of 0.70, indicating a high degree of reliability for the study’s instruments. Similarly, “composite reliability” varies between 0.816 and 0.895, also surpassing the benchmark of 0.70. These results are detailed in Table 2 and Figure 2.

**Table 2:** Constructs reliability and validity.

Construct	Cronbach’s alpha	rho_A	Composite Reliability	AVE
CA	0.854	0.860	0.891	0.578
DT	0.824	0.840	0.870	0.530
EINB	0.859	0.871	0.895	0.587
OCS	0.758	0.776	0.846	0.580
TLS	0.709	0.770	0.816	0.528

AVE, average variance extracted.

In the following stage, estimation models’ validity was surveyed with a two-advance methodology, e.g., convergency and validity of discriminant. In evaluating “convergent validity,” this study investigated the “average variance extracted (AVEs).” The findings reveal that the “AVEs” range between 0.528 and 0.587, which is deemed satisfactory and exceeds the threshold of 0.5. The results of all “validity and reliability” assessments are presented in Table 2.

#### 4.2.2 Discriminant validity

Additionally, “exploratory factor analysis (EFA)” was conducted on the study items, resulting in a solution for five factors. This solution accounted for 64.36% of the total variance, with a value of KMO equals to 0.908 and a statistically significant “Bartlett’s test” outcome (Approx. Chi-Square = 6462.633; *p* = 0.000). The scales regarding each factor exhibited strong associations within them, confirming the scales’ “convergent validity.” Moreover, the study evaluated the “Fornell-Larcker criterion” and “Heterotrait-Monotrait (HTMT)” to assess the “discriminant validity” of the models. According to the “Fornell-Larcker criterion,” the “square root of AVEs” must exceed other correlations within the variables in the same model (Hair Jr et al., 2023). The outcomes of this analysis meet this criterion, and the full assessment of the “Fornell-Larcker criterion” is provided in Table 3.

**Table 3:** Discriminant validity.

	CA	DT	EINB	OCS	TLS
Fornell-Larcker Criterion					
CA	**0.760**				
DT	0.711	**0.778**			
EINB	0.749	0.759	**0.863**		
OCS	0.711	0.728	0.766	**0.786**	
TLS	0.668	0.663	0.753	0.661	**0.726**
Heterotrait-Monotrait (HTMT) criterion					
CA					
DT	0.816				
EINB	0.812	0.825			
OCS	0.815	0.738	0.627		
TLS	0.802	0.827	0.810	0.519	

Bold and underlined Values are the square root of AVE; Heterotrait—Monotrait (HTMT) Ratio, at (HTMT < 0.85).

Furthermore, an assessment using HTMT techniques as suggested by Henseler et al. (2015) has been carried out. As indicated in Table 3, all values are below the HTMT.85 threshold, indicating that the discriminant validity met the criteria (Kline, 2015).

#### 4.2.3 Common methods bias test

Performing a “common method bias” assessment is crucial in this investigation due to the use of the same questionnaire set for collecting both dependent and external constructs’ data (Lowry and Gaskin, 2014). To evaluate the constructs of the study, “Harman’s single-factor” test has been utilized. The results indicated that the “single factor” explained around 42.45% of the “total variance.” This percentage is lower than 50%, suggesting that there is no presence of “common method bias” (Lowry and Gaskin, 2014).

#### 4.2.4 Model fit

The “standardized root mean square residual (SRMR)” criteria are employed to ensure the absence of the model’s misspecification. SRMR assesses the disparities between the anticipated correlations and observed ones. As depicted in Table 4, the SRMR value is recorded at 0.076, falling below the threshold of 0.08, which suggests a strong model fit (Henseler et al., 2016).

**Table 4:** Model fits.

	Saturated Model	Estimated Model
SRMR	0.076	0.076
d_ULS	0.979	0.979
d_G	0.307	0.307
Chi-Square	468.071	468.071
NFI	0.952	0.952

#### 4.2.5 “Predictive power” of the models

The “coefficient of determination (R^2^),” for independent and mediating constructs (DT, CA, OCS, and TLS) effectively accounts for 79.4% of the overall variance in EINB. Additionally, DT moderately explains 50.5 and 60.5% of the total variance in CA and OCS, respectively. Table 5 indicates minimal disparities between “R^2^ and adjusted R^2”^ values.

**Table 5:** R square, and R square adjusted.

	R square	R square adjusted
CA	0.505	0.503
EINB	0.794	0.790
OCS	0.605	0.604

#### 4.2.6 Multicollinearity test

The “Variance Inflation Factor (VIF)” values in Table 6 vary from 1.000 to 2.952, all falling below the critical threshold of 5. This suggests that there is no “multicollinearity” issue within the models (Lowry and Gaskin, 2014; Ringle et al., 2015).

**Table 6:** VIF values.

Items	Outer VIF values	Inner VIF values		
		EINB	CA	OCS
Employee Innovative Behavior (EINB)				
EINB1	1.686			
EINB2	1.612			
EINB3	2.130			
EINB4	1.828			
EINB5	1.948			
EINB6	1.839			
Challenging Appraisal (CA)		2.435		
CA1	1.457			
CA2	2.065			
CA3	1.992			
CA4	2.184			
CA5	1.978			
CA6	1.866			
Digital Transformation (DT)		2.952	1.000	1.000
DT1	1.544			
DT2	1.632			
DT3	1.524			
DT4	1.427			
DT5	1.669			
DT6	1.814			
Organizational Culture Support (OCS)		2.072		
OCS1	1.293			
OCS2	1.485			
OCS3	1.665			
OCS4	1.525			
Transformation Leadership Style (TLS)		2.820		
TLS1	1.316			
TLS2	1.358			
TLS3	1.271			
TLS4	1.400			

#### 4.2.7 Correlation analysis

We performed the variables’ descriptive analysis, which involved determining “means (M) and standard deviations (Std. Deviation),” as well as assessing correlations among the constructs. As shown in Table 7, all correlation coefficients are below the threshold of 0.9. This indicates the absence of common method bias among the study variables (Lowry and Gaskin, 2014). This suggests that multicollinearity is not a major issue and that the constructs in the study are statistically distinct. Table 7 provides descriptive statistics and a correlation matrix for the variables in the study namely Challenging Appraisal (CA), Digital Transformation (DT), Employee Innovative Behavior (EINB), Organizational Culture Support (OCS), and Transformational Leadership Style (TLS).

**Table 7:** Descriptives statistics and correlation matrix.

	Mean	Std. Deviation	CA	DT	EINB	OCS	TLS
CA	28.88	7.483	1.000				
DT	24.74	6.710	0.677^**^	1.000			
EINB	29.04	6.806	0.722^**^	0.733^**^	1.000		
OCS	15.16	3.821	0.683^**^	0.732^**^	0.818^**^	1.000	
TLS	18.94	3.983	0.636^**^	0.625^**^	0.687^**^	0.724^**^	1.000

** Correlation is significant at the 0.01 level (2-tailed).

Regarding descriptive statistics, we considered Mean and Standard Deviation: These columns display the average score and variability of each variable, respectively, across all respondents. On the other hand, the correlation matrix reveals the correlation coefficients between each pair of variables. Notably, Challenging Appraisal (CA) shows a moderate positive association (r = 0.677, *p* < 0.01) with Digital Transformation (DT), suggesting that as CA increases, DT tends to increase as well. This suggests that organizations investing in digital transformation tend to implement more challenging appraisal mechanisms, potentially fostering a performance-driven culture. Similarly, CA exhibits a strong and positive association (r = 0.722, *p* < 0.01) with Employee Innovative Behavior (EINB), indicating that higher CA levels are associated with greater EINB. This highlights the role of CA in promoting innovation by setting high performance expectations and encouraging creative problem-solving. Additionally, DT demonstrates a strong and positive connection (r = 0.733, *p* < 0.01) with EINB, reflecting that organizations with advanced digital transformation initiatives tend to foster higher levels of employee innovation. This finding aligns with prior research suggesting that digital advancements provide employees with tools, data-driven insights, and automation capabilities that enhance their ability to generate novel ideas. Moreover, Organizational Culture Support (OCS) exhibits a strong and positive association (r = 0.818, *p* < 0.01) with EINB, suggesting that a supportive organizational culture promotes employee innovation. A culture that encourages risk-taking, knowledge-sharing, and collaborative learning appears to enhance employees’ willingness and capacity to engage in innovative behaviors. Lastly, Transformational Leadership Style (TLS) shows a strong positive and association (r = 0.724, *p* < 0.01) with EINB, indicating that leadership styles characterized by transformational traits are associated with increased employee innovative behavior. This reinforcing the view that leaders who inspire and challenge employees contribute significantly to innovation.

#### 4.2.8 Research hypotheses

To investigate the study’s different hypotheses, we utilized “structural equation modeling (SEM)” via the statistical tool, SmartPLS (Ringle et al., 2015). The results of the distinct hypotheses evaluated and associated estimates are presented in Table 8. Specifically, the initial analysis focused on exploring the link between digital transformation (DT) and employee innovative behavior (EINB). The result reveals that DT positively influences EINB (DT → EINB, β = 0.130, t = 2.321, *p* < 0.05). This suggests that for every incremental increase in digital transformation, there is a corresponding 13% increase in EINB, with all other variables held constant. Consequently, hypothesis 1 (H1) is validated. While digital transformation is positively associated with innovation, other underlying mechanisms may be at play, such as employee autonomy, digital literacy, or external market pressures. Future studies could examine whether digital transformation’s impact on innovation varies across industries with differing levels of technological maturity.

**Table 8:** Hypothesis test.

Hypothesis	Path	Coef.	St. Error	t-stat	*p*-value	Decision
H1	DT → EINB	0.130	0.056	2.321	0.021	Confirmed
H2	DT → CA	0.711	0.033	21.838	0.000	Confirmed
H3	DT → OCS	0.778	0.018	42.883	0.000	Confirmed
H4	CA → EINB	0.207	0.044	4.730	0.000	Confirmed
H5	OCS → EINB	0.517	0.065	7.926	0.000	Confirmed
H6	TLS → EINB	0.114	0.058	1.920	0.036	Confirmed
H7	DT*TLS → EINB	−0.025	0.068	0.375	0.708	Not confirmed
H8	DT → CA → EINB	0.147	0.030	4.890	0.000	Confirmed
H9	DT → OCS → EINB	0.403	0.052	7.788	0.000	Confirmed

In relation to the second hypothesis, the examination indicates a significant and positive linkage between digital transformation and CA (DT → CA, β = 0.711, t = 21.838, *p* < 0.05). This suggests that a one-unit increased in digital transformation while controlling for other factors, leads to a 71.1% increase in CA. Consequently, hypothesis 2 (H2) is affirmed. This suggests that as organizations integrate digital tools, they can also implement more sophisticated performance appraisal systems, potentially using AI-driven analytics to track and evaluate employee performance dynamically. Similarly, hypothesis 3 linked DT and OCS, and the results reveal that digital transformation influences positively organizational culture support (DT → OCS, β = 0.778, t = 42.883, *p* < 0.05). This outcome supports hypothesis 3 (H3). This further implies that digital transformation may foster a more adaptive and resilient organizational culture, characterized by knowledge-sharing, flexibility, and a data-driven decision-making approach. Another result indicates a significant and positive impact of challenging appraisal (CA) on EINB (CA → EINB, β = 0.207, t = 4.730, *p* < 0.05). This demonstrates that for each unit increase in challenging appraisal, there is a 20.7% increase in EINB. As a result, hypothesis 4 (H4) is supported. This highlights that performance assessments that set ambitious goals and encourage problem-solving can drive employees to adopt more innovative approaches in their work. In addition, Hypotheses 5 (OCS → EINB, β = 0.517, t = 7.926, *p* < 0.05), and 6 (TLS → EINB, β = 0.114, t = 1.92, *p* < 0.05) are also supported, indicating that Organizational Culture Support and “Transformational Leadership Style” have significant positive effects on Employee Innovative Behavior. The stronger effect of OCS (β = 0.517) compared to TLS (β = 0.114) suggests that a supportive culture may be more influential than leadership alone in driving innovation. On the other hand, hypothesis 7 tests the interaction effect between Digital Transformation and Transformational Leadership Style (DT*TLS) on Employee Innovative Behavior. However, the result is not significant (*p* > 0.05), indicating that the interaction between DT and TLS does not have a significant effect on EINB. The non-significant interaction between DT and TLS suggests that leadership alone may not amplify digital transformation’s effect on employee innovation. It is possible that employees require additional factors such as training, incentives, or psychological safety to fully harness the benefits of digital tools.

Finally, the mediating effects of challenging appraisal and organizational culture support are further assessed on the link between DT and EINB. Hypotheses 8 and 9 examine the mediated relationships between variables. Both Hypothesis 8 (DT → CA → EINB, β = 0.147, t = 4.890, *p* < 0.05) and Hypothesis 9 (DT → OCS → EINB, β = 0.403, t = 7.788, *p* < 0.05) are supported, indicating that Challenging Appraisal and Organizational Culture Support positively and significantly mediate the link between Digital Transformation and Employee Innovative Behavior. This suggests that digital transformation alone may not be enough to drive innovation unless accompanied by appraisal mechanisms that challenge employees to exceed expectations. This finding reinforces the importance of fostering a work environment that embraces change, experimentation, and knowledge-sharing to maximize the benefits of digital transformation on innovation. All bootstrapping outcomes are depicted in Table 8.

**Figure 3:**
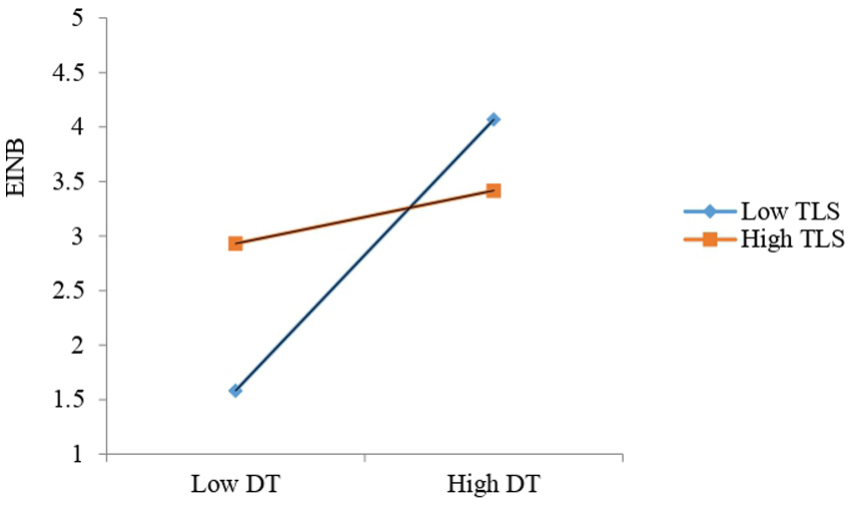
Interaction effect of TLS on DT-EINB.

It is important to notice that TLS dampens the positive relationship between DT and EINB (see Figure 3). This implies that the higher the transformation leadership style (TLS), the weaker the link between digital transformation (DT) and employee innovative behavior (EINB). In other words, the lower the TLS, the greater the interaction effect and the stronger the link between DT and EINB.

Based the findings, the following direct implications are highlighted. First, businesses aiming to enhance innovation through digital transformation should simultaneously focus on strengthening their appraisal systems and fostering a culture of support. Digital tools should be integrated with clear performance expectations and feedback mechanisms to maximize innovation potential. Second, transformational leadership, while important, may need to be complemented with specific skill development and autonomy-enhancing policies to empower employees to leverage digital transformation effectively. Finally, organizations undergoing digital transformation should consider policies that promote a balance between technological advancements and human-centered workplace strategies. Investment in digital literacy and change management programs could be key to ensuring successful innovation outcomes.

Overall, the study findings reinforce the notion that digital transformation serves as a critical enabler of employee innovation, particularly when supported by challenging appraisal systems and a strong organizational culture. While transformational leadership is beneficial, its role in amplifying digital transformation’s effect on innovation remains unclear. Future research should explore industry-specific variations and additional mediating factors such as psychological safety, digital literacy, and job autonomy to provide a more comprehensive understanding of these dynamics.

## 5 Discussion

The findings of the study, analyzed through structural equation modeling (SEM) utilizing SmartPLS, shed light on the complex interplay between digital transformation (DT), challenging appraisal (CA), organizational culture support (OCS), transformational leadership style (TLS), and employee innovative behavior (EINB).

Firstly, the analysis demonstrated a significant positive linkage between digital transformation and employee innovative behavior (H1). The findings suggest that organizations undergoing digital transformation experience higher levels of innovative behavior among employees, indicating that the adoption of digital technologies positively influences the generation of new ideas and solutions within the organizational context. Empirical evidence supporting the positive link between digital transformation and EINB is abundant. Studies by Bharadwaj et al. (2013) and Iivari et al. (2020) have demonstrated that digital transformation initiatives equip employees with enhanced access to data and information, fostering idea generation, problem-solving, and knowledge-sharing, thereby cultivating a culture of innovation. Similarly, research by Galliers and Leidner (2014) and Scuotto et al. (2023) underscores the role of employees’ adaptability to technological changes induced by digital transformation in promoting innovative behaviors. Moreover, Huu (2023) highlighted the positive association between the use of digital technologies for problem-solving purposes and employee innovative behavior. Our study’s confirmation of Hypothesis 1 further strengthens this body of evidence, indicating that digital transformation indeed positively influences employee innovative behavior.

Secondly, the study found that digital transformation significantly predicts challenging appraisal practices (H2). This indicates that as organizations embrace digital transformation initiatives, they are more likely to adopt challenging appraisal processes that focus on continuous improvement, feedback, and development rather than mere assessment. Regarding the relationship between digital transformation and challenging appraisal, empirical studies have provided insights into how digital transformation initiatives impact organizational culture and appraisal practices. Research by Saeed et al. (2023) and Trenerry et al. (2021) suggests that digital transformation fosters a culture of agility, experimentation, and continuous improvement, aligning well with challenging appraisal practices. Mihai et al. (2022) also highlighted how digital transformation enables the implementation of real-time performance metrics and feedback mechanisms, contributing to more dynamic and personalized appraisal systems. The findings of our study, confirming Hypothesis 2, further support these assertions, indicating that organizations undergoing digital transformation are more likely to adopt challenging appraisal practices.

Similarly, the analysis confirms the positive influence of digital transformation on organizational culture support (H3). This suggests that organizations undergoing digital transformation are likely to foster supportive organizational cultures. This finding aligns with studies by Nazir et al. (2019), which highlighted the role of digital transformation initiatives in shaping supportive organizational cultures. Nonetheless, Ghafoori et al. (2024) introduce nuances to this relationship, suggesting that contextual factors may moderate the impact of digital transformation on organizational culture support.

Moreover, the analysis demonstrated a significant positive influence of challenging appraisal on employee innovative behavior (H4). Employees who receive constructive feedback, set challenging yet achievable goals, and engage in ongoing skill development are more likely to exhibit innovative behaviors, contributing to a culture of creativity and problem-solving within the organization. Furthermore, empirical evidence has consistently demonstrated the positive influence of challenging appraisal on employee innovative behavior. Studies by Ashford et al. (2016), Miao et al. (2023), and Zhou et al. (2014) emphasize how high-quality feedback, growth-oriented mindsets, and recognition of risk-taking behavior inherent in challenging appraisal processes stimulate employee creativity and innovation. Van Dyck et al. (2005) also suggested that an organizational culture emphasizing frequent and challenging feedback fosters an environment conducive to idea generation. Our study’s confirmation of Hypothesis 4 adds to this body of literature, reaffirming that challenging appraisal indeed plays a vital role in fostering employee innovative behavior.

Both organizational culture support and transformational leadership style are shown to have significant positive effects on employee innovative behavior. This suggests that supportive organizational cultures and transformational leadership styles contribute to fostering innovation among employees. This finding aligns with studies by Jiang and Chen (2018), which emphasized the importance of supportive organizational cultures and transformational leadership styles in promoting innovation. Yet, Saeed et al. (2023) suggest that the direct impact of these factors on innovative behavior may vary depending on contextual factors.

The interaction effect between digital transformation and transformational leadership style on employee innovative behavior is found to be non-significant. This implies that the linkage between digital transformation and employee innovative behavior is not significantly influenced by transformational leadership style. While not directly conflicting, Trenerry et al. (2021) suggest that the interaction effect between digital transformation and transformational leadership style may be contingent on organizational contexts and employee perceptions.

Lastly, this study also tests the mediating effects of challenging appraisal and organizational culture support in the linkage between digital transformation and employee innovative behavior, finding significant mediation effects. This indicates that challenging appraisal and organizational culture support play important intermediary roles in translating digital transformation efforts into employee outcomes (H8 and H9). This suggests that challenging appraisal and organizational culture support play a crucial role in translating the impact of digital transformation initiatives into tangible improvements in innovative behavior among employees. These results are in line with studies by Zhang G. et al. (2023) and Liu and Su (2022) which demonstrated the mediating effects of challenging appraisal and organizational culture support in facilitating the link between digital transformation and employee innovative behavior. However, Chen et al. (2024) introduce complexities to this relationship, suggesting that mediating effects may be influenced by contextual factors. Moreover, emerging research by Westerman et al. (2014), (Scuotto et al., 2023), and AlNuaimi et al. (2022) hints at the potential mediating effect of challenging appraisal practices. These studies suggest that challenging appraisal practices complement digital transformation initiatives by fostering a culture of agility, learning, and experimentation, thereby influencing employee behaviors and potentially mediating the influence of digital transformation on innovation.

The findings of this study align with existing empirical evidence highlighting the importance of digital transformation and challenging appraisal practices in fostering employee innovative behavior within organizations. The results underscore the need for organizations to not only invest in digital technologies but also cultivate a supportive culture that encourages continuous learning, feedback, and experimentation to drive innovation. In conclusion, our investigation contributes to the growing body of literature on the interplay between digital transformation, challenging appraisal, and employee innovative behavior. By integrating empirical evidence from existing studies, we provide a comprehensive understanding of how these factors influence organizational innovation dynamics. However, further research is warranted to explore additional contextual factors and mechanisms that may influence these relationships, thereby enhancing our understanding of organizational innovation processes in the digital age.

### 5.1 Conclusion

This study investigated the influence of digital transformation on employee innovative behavior, with a focus on the mediating roles of challenging appraisal and organizational culture support, as well as the moderating effect of transformational leadership style. The survey data collected from employees of the top five banks in Congo Brazzaville provided valuable insights into these relationships’ complex dynamics. By drawing upon Social Cognitive Theory and Self-determination Theory, the study elucidated the mechanisms through which digital transformation initiatives shape employee behavior and organizational outcomes.

The findings of the study, analyzed using Structural Equation Modeling (SEM) via SmartPLS, revealed several important insights. Firstly, digital transformation was found to have a significant positive influence on employee innovative behavior, highlighting the importance of organizations embracing technological advancements to foster innovation among their workforce. Secondly, challenging appraisal and organizational culture support were identified as key mediating factors in translating digital transformation efforts into tangible outcomes, emphasizing the role of organizational practices in shaping employee responses to change. Thirdly, transformational leadership style was found to have a significant positive effect on employee innovative behavior, although its interaction with digital transformation did not yield significant results, suggesting the need for further exploration of contextual factors. These results carry significant implications for both theoretical understanding and practical application. From a theoretical perspective, the study contributes to our understanding of the mechanisms through which digital transformation initiatives influence employee behavior, providing empirical support for the theories of Social Cognitive and Self-determination. By integrating these theoretical frameworks, the study offers a comprehensive perspective on the interplay between individual, organizational, and environmental factors in shaping employee responses to digital transformation. The study also contributes to the broader digital transformation literature by offering a nuanced perspective on the roles of appraisal and cultural support in fostering innovation within banking institutions.

From a practical standpoint, the findings underscore the importance of organizational practices in fostering innovation in the digital age. Particularly, the findings provide several actionable insights for organizations aiming to maximize the benefits of digital transformation. First, banks and financial institutions should invest in digital upskilling programs to ensure that employees possess the necessary technical and cognitive skills to innovate effectively in a digitally driven workplace. Second, organizations should implement performance management systems that incorporate challenging appraisal mechanisms, as they encourage employees to develop problem-solving skills, embrace new technologies, and proactively contribute to digital initiatives. Third, fostering a supportive organizational culture that encourages collaboration, risk-taking, and openness to change is critical in sustaining digital transformation efforts. Leaders and managers must prioritize cultural transformation alongside technological advancements to ensure employees feel engaged and empowered to contribute to digital innovation. Furthermore, the study highlights the need for targeted leadership development programs to strengthen transformational leadership skills, as such leadership plays a vital role in motivating employees, fostering a vision for digital change, and reducing resistance to new technologies. Organizations should integrate leadership strategies that emphasize continuous learning, adaptive management, and inclusive decision-making to enhance the effectiveness of digital transformation initiatives. In addition to organizational implications, the findings also hold broader societal relevance. By successfully integrating digital transformation strategies, banks can enhance financial inclusion, streamline service delivery, and improve overall customer experiences. This, in turn, fosters economic growth by expanding access to financial services, supporting entrepreneurship, and driving technological adoption within the broader economy. Policymakers and industry regulators should develop supportive policies that incentivize banks to invest in employee digital training and innovation-friendly work environments, ensuring that digital transformation benefits both employees and society at large.

Overall, this work contributes to the growing body of literature on digital transformation and employee behavior by providing empirical evidence of the mechanisms underlying these relationships. By leveraging insights from Social Cognitive and Self-determination Theory, the study highlights the significance of digital transformation and challenging appraisal practices in fostering employee innovative behavior, offering valuable implications for organizational management and strategic decision-making in the era of digitalization.

### 5.2 Implications of the study

This study provides significant implications for both the banking sector and society by demonstrating how digital transformation fosters employee innovative behavior, ultimately driving efficiency, competitiveness, and long-term sustainability. In an era where digitalization is reshaping financial services, banks must actively invest in advanced digital tools, automation, and artificial intelligence to streamline operations, enhance decision-making, and improve customer experiences. Equally important is the need for comprehensive training programs that equip employees with the necessary digital skills to adapt to evolving technologies and leverage them for innovation. Additionally, organizations should implement challenging appraisal systems that encourage continuous learning, problem-solving, and creativity among employees, ensuring that performance evaluation frameworks are designed to stimulate rather than hinder innovation. Moreover, fostering a supportive organizational culture that values collaboration, openness to new ideas, and risk-taking is essential for sustaining digital transformation efforts. Leadership plays a pivotal role in this process, as transformational leaders can inspire and guide employees through technological transitions by providing vision, motivation, and mentorship. Organizations should develop leadership programs that enhance transformational leadership skills, enabling leaders to support their teams effectively and create an environment conducive to innovation. Beyond the organizational level, digital transformation in banking has broader societal implications. By increasing operational efficiency and improving service delivery, banks can expand financial inclusion, particularly in underserved areas, bridging the gap between traditional banking systems and digital financial services. This, in turn, contributes to economic growth by fostering financial stability, increasing access to credit, and supporting entrepreneurship. Additionally, workforce development is strengthened as employees gain digital competencies that enhance their employability and adaptability in an increasingly technology-driven job market.

From a policy perspective, governments and regulatory bodies should create supportive frameworks that encourage digital adoption while ensuring job security and employee well-being. Policies should promote incentives for digital upskilling, encourage banks to invest in employee development, and introduce regulations that balance technological advancement with ethical considerations. Furthermore, leadership development programs should be integrated into organizational strategies to cultivate an innovation-driven workplace where employees feel empowered to experiment, take initiative, and contribute to organizational success.

All in all, integrating these insights can help banks, industry leaders, and policymakers develop a holistic and sustainable approach to digital transformation, maximizing its benefits while mitigating potential challenges. By embracing digitalization strategically, organizations can enhance both employee innovative behavior and overall performance, ultimately contributing to a more resilient and dynamic financial sector that positively impacts society.

## 6 Limitations and further research directions

While offering valuable insights, this study has limitations that merit consideration. Firstly, its quantitative approach may not fully capture the intricate relationships between digital transformation, challenging appraisal, organizational culture support, transformational leadership style, and employee innovative behavior. Future research could employ qualitative methods like interviews or focus groups for a deeper understanding. Secondly, while the sample size of 280 respondents was randomly selected, it may not fully represent all employees in Congolese banks. Selection bias may be present if respondents who are more engaged with digital transformation initiatives were more likely to participate, potentially skewing results. Future studies should aim for larger and more diverse sample sizes to enhance generalizability and reduce selection bias. Expanding the study to include a more representative mix of employees across different hierarchical levels, departments, and banking institutions would provide a more holistic view of digital transformation’s impact. Moreover, focusing solely on Congolese banks limits the findings’ applicability to other industries or regions. The banking sector has unique regulatory, technological, and operational characteristics that may not be directly comparable to other sectors. Additionally, cultural and economic differences across countries may influence how digital transformation and leadership styles interact with employee behavior. Future research should explore these dynamics in diverse organizational and national contexts, particularly in industries with varying levels of technological integration. A comparative analysis across multiple industries and countries would provide valuable cross-sectoral and cross-cultural insights. Future studies should explore these dynamics in diverse contexts. Furthermore, reliance on self-reported questionnaire data may introduce response biases. Using objective measures or behavioral observations could provide more accurate assessments. Moreover, the study identifies significant relationships, but their directionality remains unclear, as digital transformation may both influence and be influenced by employee innovative behavior. Future longitudinal studies tracking changes over time would offer stronger evidence of causality and allow researchers to assess the long-term impact of digital transformation on employee innovation and organizational performance.

Moving forward, research should examine contextual factors’ moderating effects, such as organizational culture and leadership style, on digital transformation and challenging appraisal. Understanding how individual differences influence responses to these practices is crucial, as is exploring cultural variations across different regions. Moreover, future studies should incorporate additional mediators and moderators to deepen understanding. Finally, longitudinal research on the long-term effects of digital transformation and challenging appraisal on organizational performance outcomes would offer strategic insights into their sustainability and impact. Investigating whether sustained digital transformation efforts lead to long-term cultural shifts within organizations or if their effects diminish over time would be particularly valuable for both academic research and managerial practice. By addressing these limitations and expanding research directions, future studies can build on the findings of this study to enhance theoretical and practical understanding of digital transformation in the evolving workplace.

## Data Availability

The raw data supporting the conclusions of this article will be made available by the authors, without undue reservation.
