# Supplementary material for: Influence of digital transformation on employee innovative behavior: roles of challenging appraisal, organizational culture support, and transformational leadership style
Source: Front Psychol. 2025 Mar 19;16:1532977. doi: 10.3389/fpsyg.2025.1532977 (PMC11963808; doi:10.3389/fpsyg.2025.1532977)
Supplement: Supplementary file 1 [file Supplementary_file_1.docx]

**APPENDIX A: Study Survey Questionnaires**

| **Part I: Demographic information. Please tick the option or box that best describes your answer to each question.** | | |
| --- | --- | --- |
| **1. Age:** | □ 24 - 29  □ 30 - 35 | □ 36 - 40  □ 41 and above |
| **2. Gender:** | □ Male | □ Female |
| **3. Work Experience:** | □ less and equal 3  □ 4 - 6 | □ 7 - 9  □ 10 and higher |
| **4 Field or Department:** | □ Finance  □ Marketing | □ Customer service  □ Human Resource |
| **6. Education Level:** | □ Bachelor/Undergraduate  □ Graduates (Master or Ph.D.) | □ Others |

**Note: Please tick the option or number from 1 to 5 based on your experience with your job and according to the related statement of each question.** Here 1 means strongly disagree (SD), 2 means disagree (D), 3 means neutral (N), 4 means agree (A) and 5 means strongly agree (SA).

**Part II: Dital Transformation**

| **Construct** | **Code** | **Questionnaire Item** | **1** | **2** | **3** | **4** | **5** |
| --- | --- | --- | --- | --- | --- | --- | --- |
| Dital Transformation (DT) | DT1 | The features of our digital solutions are superior compared to our competitors. |  |  |  |  |  |
|  | DT2 | The quality of our digital solutions is superior compared to our competitors. |  |  |  |  |  |
|  | DT3 | Our digital solutions are different from our competitors’ in terms of product platform. |  |  |  |  |  |
|  | DT4 | The applications of our digital solutions are totally different from our competitors. |  |  |  |  |  |
|  | DT5 | Some of our digital solutions are new to the market at the time of launching. |  |  |  |  |  |
|  | DT6 | Our new digital solutions are minor improvements of existing products. |  |  |  |  |  |

**Part III: Challenging Appraisal**

| **Construct** | **Code** | **Questionnaire Item** | **1** | **2** | **3** | **4** | **5** |
| --- | --- | --- | --- | --- | --- | --- | --- |
| Challenging Appraisal (CA) | CA1 | The number of projects and/or assignments I have. |  |  |  |  |  |
|  | CA2 | The amount of time I spend at work. |  |  |  |  |  |
|  | CA3 | The volume of work that must be accomplished in the allotted time |  |  |  |  |  |
|  | CA4 | Time pressures I experience |  |  |  |  |  |
|  | CA5 | The amount of responsibility I have |  |  |  |  |  |
|  | CA6 | The scope of responsibility my position entails. |  |  |  |  |  |

**Part IV: Organizational Culture Support**

| **Construct** | **Code** | **Questionnaire Item** | **1** | **2** | **3** | **4** | **5** |
| --- | --- | --- | --- | --- | --- | --- | --- |
| Organizational Culture Support (OCS) | OCS1 | This company encourages me to exert my utmost efforts |  |  |  |  |  |
|  | OCS2 | This company maintains consistency and fairness in its treatment of people. |  |  |  |  |  |
|  | OCS3 | This company appreciates the input of employees at all levels. |  |  |  |  |  |
|  | OCS4 | I am encouraged to provide recommendations for improving my job performance |  |  |  |  |  |

**Part V: Transformation Leadership Style**

| **Construct** | **Code** | **Questionnaire Item** | **1** | **2** | **3** | **4** | **5** |
| --- | --- | --- | --- | --- | --- | --- | --- |
| Transformational Leadership Style (TLS) | TLS1 | My leader persuades me to prioritize the collective good over individual interests |  |  |  |  |  |
|  | TLS2 | My leader proposes alternative perspectives on task completion strategies. |  |  |  |  |  |
|  | TLS3 | My leader dedicates time to mentoring, teaching, and coaching their followers. |  |  |  |  |  |
|  | TLS4 | My leader assists others in nurturing their strengths. |  |  |  |  |  |

**Part VI: Employee Innovative Behavior**

| **Construct** | **Code** | **Questionnaire Item** | 1 | 2 | 3 | 4 | 5 |
| --- | --- | --- | --- | --- | --- | --- | --- |
| Employee Innovative Behavior (EINB) | EINB1 | Our ability to function creatively is respected by the leadership. |  |  |  |  |  |
|  | EINB2 | Creativity is encouraged here |  |  |  |  |  |
|  | EINB3 | This organization gives me free time to pursue creative ideas  during the workday. |  |  |  |  |  |
|  | EINB4 | There are adequate resources devoted to innovation in this  organization. |  |  |  |  |  |
|  | EINB5 | The reward system here encourages innovation. |  |  |  |  |  |
|  | EINB6 | I am creative at work. |  |  |  |  |  |
